# Liver and Pancreatic Toxicity of Endocrine-Disruptive Chemicals: Focus on Mitochondrial Dysfunction and Oxidative Stress

**DOI:** 10.3390/ijms25137420

**Published:** 2024-07-06

**Authors:** Adina V. Lința, Bogdan M. Lolescu, Cosmin A. Ilie, Mihaela Vlad, Alexandru Blidișel, Adrian Sturza, Claudia Borza, Danina M. Muntean, Octavian M. Crețu

**Affiliations:** 1 Department of Functional Sciences—Chair of Pathophysiology, “Victor Babeș” University of Medicine and Pharmacy of Timișoara, E. Murgu Sq. No. 2, 300041 Timișoara, Romania; adina.linta@umft.ro (A.V.L.); sturza.adrian@umft.ro (A.S.); borza.claudia@umft.ro (C.B.); 2 Centre for Translational Research and Systems Medicine, “Victor Babeș” University of Medicine and Pharmacy of Timișoara, E. Murgu Sq. No. 2, 300041 Timișoara, Romania; bogdan.lolescu@umft.ro (B.M.L.); ilie.adrian@umft.ro (C.A.I.); 3 Doctoral School Medicine-Pharmacy, “Victor Babeș” University of Medicine and Pharmacy of Timișoara, E. Murgu Sq., No. 2, 300041 Timișoara, Romania; 4 Department of Functional Sciences—Chair of Public Health & Sanitary Management, “Victor Babeș” University of Medicine and Pharmacy of Timișoara, E. Murgu Sq. No. 2, 300041 Timișoara, Romania; 5 Department of Internal Medicine II—Chair of Endocrinology, “Victor Babeș” University of Medicine and Pharmacy of Timișoara, E. Murgu Sq., No. 2, 300041 Timișoara, Romania; vlad.mihaela@umft.ro; 6 Department of Surgery I—Chair of Surgical Semiotics & Thoracic Surgery, “Victor Babeș” University of Medicine and Pharmacy of Timișoara, E. Murgu Sq. No. 2, 300041 Timişoara, Romania; blidy@umft.ro (A.B.); octavian.cretu@umft.ro (O.M.C.); 7 Centre for Hepato-Biliary and Pancreatic Surgery, “Victor Babeș” University of Medicine and Pharmacy of Timișoara, E. Murgu Sq. No. 2, 300041 Timişoara, Romania

**Keywords:** endocrine disruptors, metabolism-disrupting chemicals, liver, pancreas, mitochondrial dysfunction, oxidative stress

## Abstract

In recent years, the worldwide epidemic of metabolic diseases, namely obesity, metabolic syndrome, diabetes and metabolic-associated fatty liver disease (MAFLD) has been strongly associated with constant exposure to endocrine-disruptive chemicals (EDCs), in particular, the ones able to disrupt various metabolic pathways. EDCs have a negative impact on several human tissues/systems, including metabolically active organs, such as the liver and pancreas. Among their deleterious effects, EDCs induce mitochondrial dysfunction and oxidative stress, which are also the major pathophysiological mechanisms underlying metabolic diseases. In this narrative review, we delve into the current literature on EDC toxicity effects on the liver and pancreatic tissues in terms of impaired mitochondrial function and redox homeostasis.

## 1. Introduction

The global rates of obesity/overweight and their cardio-metabolic complications have risen dramatically during the past 3 decades. These conditions rank high among the leading causes of death worldwide, and in order to fight this global health crisis, a thorough understanding of their pathophysiology is still required [1,2].

Several factors variably contribute to the development of metabolic diseases, such as increased calorie intake, sedentary behavior, genetic predisposition and aging [3]. However, the available literature indicates that the worldwide increased prevalence of obesity, metabolic syndrome, diabetes, MAFLD and fatty pancreas disease cannot be entirely attributed to the previously mentioned factors. This is highlighted by the fact that, even though the calorie intake and exercise levels are almost the same, people tend to weigh more than they did 20–30 years ago [4,5].

In the past decade, environmental factors have received a well-deserved increase in attention due to the parallel rise of both metabolic pathologies and the production/usage of endocrine-disruptive chemicals [6,7,8].

The term “endocrine-disruptor” was introduced back in 1991 [9] to recognize the ability of certain environmental chemicals that interfere with hormone systems in both humans and wildlife. This concept gained prominence since studies accumulated evidence linking these compounds to various deleterious health implications, among which reproductive disorders, neurodevelopmental abnormalities and metabolic dysregulation were mostly investigated. As for the latter, a couple of years later, the term “obesogen” was coined by researchers, drawing attention to the role of EDCs in the development of obesity [10]. Obesogens are environmental agents that directly promote fat accumulation by increasing the number of adipocytes or indirectly through changes in metabolism, such as shifting energy balance to favor calorie storage over energy expenditure [10,11,12]. Building on this foundation, the concept of “metabolic endocrine disruptors or metabolism-disrupting chemicals (MDC)” has emerged to encompass a broader range of EDCs implicated not only in obesity but also in the pathogenesis of all metabolic disorders by interfering with the metabolism of the organs (liver, pancreas and adipose tissue) that are dysfunctional in these pathologies [13,14,15,16]. As such, several recent studies have highlighted the contribution of EDCs in the onset and progression of diabetes [17,18], MAFLD and pancreatic diseases [14,19].

Mitochondria are particularly vulnerable to the detrimental effects of exposure to environmental toxicants. Accordingly, EDCs have been reported to: impair the function of the mitochondrial electron transport system (ETS), lower ATP levels, cause DNA damage, interfere with the repair pathways, increase circulating cell-free mtDNA, and induce morphological alterations, such as mitochondrial fragmentation, ultimately resulting in cell death (for reviews see refs. [20,21,22]). Cumulative evidence has indicated that long-term exposure to pesticides and insecticides (organophosphates, carbamate, mancozeb, etc.) elicited abnormal mitochondrial distribution and dysfunction and negatively impacted female fertility, reproductive health, development of fetuses and endocrine function (recently reviewed in refs. [23,24,25]).

This narrative review aimed to summarize the available data regarding mitochondrial dysfunction and oxidative stress elicited by the most common MDCs on the liver and pancreas, two target organs in metabolic disorders. More precisely, we delved into the literature tackling the effects of the following ubiquitous classes of MDCs (most of them also reported as obesogens): bisphenols, alkylphenols, polychlorinated biphenyls, triazines, phthalates, per- and polyfluoroalkyl substances, polychlorinated diphenyl ethers and organotins on liver and pancreatic mitochondrial dysfunction and oxidative stress.

## 2. Data Sources

We searched the PubMed and Web of Science databases using the following terms: “endocrine disruptors”, “metabolism-disrupting chemicals”, “bisphenols”, “polychlorinated biphenyls”, “atrazine”, “phthalates”, “perfluorooctane sulfonate”, “polychlorinated diphenyl ethers”, “tributyltin”, “toxicity”, “mitochondria”, “oxidative stress”, “liver”, “liver cells”, “pancreas” and “pancreatic cells”. Relevant full-text articles, including experimental research (mainly), clinical trials, epidemiological evidence and reviews related to the topic, were screened, and the relevant ones published in English up to April 2024 were included.

## 3. Mitochondrial Dysfunction and Oxidative Stress Are Common Pathomechanisms of EDC Toxicity and Metabolic Pathologies

Mitochondria are unique, multifunctional and malleable organelles with organ-related complex phenotypes and heterogeneity [26]. In addition to their primary role in energy metabolism, mitochondria are currently viewed as cellular “microprocessors” due to their critical, interconnected roles in cellular homeostasis and survival by controlling intermediary metabolism, calcium movement and storage, a plethora of signaling pathways and most mechanisms of cell death through a dynamic, network-based adaptation [27,28]. Moreover, mitochondria are both major sources and targets of reactive oxygen species (ROS) [29], thus being particularly prone to damage in the vast majority of chronic diseases [30,31,32,33,34]. Excessive ROS generation will alter mitochondrial membrane permeability, impair respiration, elicit structural damage and cause mutations in mitochondrial DNA (mtDNA), whose potential for repair is lower than that of nuclear DNA [35,36,37,38].

Importantly, since mitochondrial dysfunction and oxidative stress are also the major pathophysiological mechanisms underlying all metabolic pathologies, the progression of these diseases and/or the occurrence of complications might be potentiated by exposure to environmental toxicants. Indeed, it has been recently hypothesized that it is the environmental chemicals (e.g., food additives) that move throughout the body and inaccurately communicate with the organs by generating misleading signals regarding the energy status, ultimately leading to obesity [39]. In a very recent, hypothesis-generating and predictive landmark paper, the group of Barbara Corkey proposed a unifying theory for the obesity pandemic (“globesity”), which posits the combined role of obesogens (mainly, bisphenol A, phthalates and polyfluorinated substances) and impaired redox homeostasis in altering metabolism via ROS-mediated signals. Accordingly, an integrated obesogene/redox model was postulated in which obesogenes/MDCs (present in food/food packaging and household products) generate false endocrine/autocrine metabolic signals via increasing ROS and altering hormone signaling. The weight gain is further mediated via the following well-described pathways: (i) increased caloric/overeating and altered hypothalamic satiety and appetite regulation (the energy balance model of obesity) and (ii) abnormal fat storage (increased cell size, number, function and growth) due to high insulin secretion/insulin resistance, low-grade inflammation and impaired microbiome (the carbohydrate-insulin model of obesity). Last but not least, if exposure to obesogens occurs in utero and/or early life, the programming of metabolism will be impaired, and so will the gene expression in the liver, adipose tissue, pancreas and brain, thus facilitating weight gain [40].

## 4. EDCs and Liver Toxicity

The liver is the central organ in human metabolism, being involved in the regulation of plasma levels of carbohydrates, lipoproteins and amino acids, storage of glycogen and triglycerides, secretion of hepatokines and modulation of peripheral insulin during the complex coordination of the body response to nutrient abundance or deficit.

There is a plethora of evidence linking pesticide exposure to liver toxicity. The drive for high crop yields has led to the misuse of pesticides, adversely affecting both ecosystems and human health. Specifically, chlorpyrifos, an organophosphate insecticide widely used for its cost-effectiveness and efficiency, has been highly researched for its liver toxicity. Excessive and unguided application of chlorpyrifos by farmers has resulted in its accumulation in crops and contamination of water sources, posing serious health risks. As such, research evaluating the toxicological effects of chlorpyrifos on rat liver showed that exposure to various levels (2.5, 5 and 10 mg/kg) led to oxidative stress via the activation of the JAK/STAT and MAPK pathways and subsequent exacerbation of inflammation and apoptosis [41]. More recently, Han et al. shed further light on the mechanism responsible for liver injury caused by chlorpyrifos and reported that mitochondrial ROS were responsible for the P53-activation of ferroptosis [42].

However, the impact of other EDCs on liver tissue has been increasingly investigated lately since there is mounting evidence that they significantly contribute to metabolic disease [4,15,43]. Among the most important chemical compounds reported to act as MDCs are the following: bisphenols, nonylphenol, polychlorinated biphenyls, atrazine, phthalates, perfluoroalkyl and polyfluoroalkyl substances and polybrominated diphenyl ethers [44].

### 4.1. Bisphenols (BPs)

Also known as the “everywhere chemicals”, strongly highlighting their omnipresence, BPs represent a large class of chemical compounds widely used for manufacturing polycarbonate plastics and epoxy resins [45], reported to cause complex organellar stress and aberrant changes in cellular signaling pathways [46].

The European Food Safety Authority and the US Environmental Protection Agency determined a tolerable daily intake (TDI) or reference dose of 0.05 mg/kg body weight (bw)/day based on rodent studies. The US Food and Drug Administration has declared a no observed adverse effect level (NOAEL) of 5 mg BPA/kg/day.

Numerous studies have proven the harmful effects of bisphenols, particularly of bisphenol A (BPA), on liver cells. According to the in vivo evidence, BPA can cause liver oxidative damage following prolonged and repetitive exposure [47]. A pioneering study back in 2008 reported the association between urinary BPA and clinically abnormal levels of two liver enzymes (gamma-glutamyltransferase-γGT, and alkaline phosphatase - ALP) as markers of liver damage, as well as the presence of diabetes [48]. These early findings were confirmed in the next years by several cross-sectional epidemiological studies reporting that individuals with high BPA concentrations in urine are more likely to suffer from diabetes, obesity and hypertension than those with low BPA in urine [49].

Given the wide range of human exposure to BPA, several experimental studies were designed to address the mechanisms of its dose-dependent toxicity. Thus, Huc et al. exposed the human liver cell line HepG2 to low concentrations of BPA ($10^{-4}-10^{-12}$ M) and assessed several parameters by flow cytometry [50]. They demonstrated that acute incubation with BPA elicited a mitochondrial dysfunction characterized by significant ROS production, hyperpolarization of mitochondrial membrane potential (MMP), lipid accumulation and release of pro-inflammatory cytokines; these authors speculated that all three pathophysiological mechanisms, i.e., mitochondrial dysfunction, impairment of lipid metabolism and inflammation, may cause liver steatosis in patients [50]. Moon et al. further confirmed that both acute and chronic exposure to BPA below the NOAEL resulted in liver mitochondrial dysfunction, increased oxidative stress and inflammation [51]. The findings of augmented oxidative stress and decreased concentration of antioxidants due to chronic exposure to BPA in different concentrations were reported by several studies conducted in murine liver tissues [52,53,54,55,56,57], as summarized in Table 1.

Pirozzi et al. examined the harmful effects of BPA on adult obese mice [58]. Following 12 weeks of high-fat diet (HFD) feeding, male C57Bl/6J mice were administered BPA orally (at a dose of 50 µg/kg) daily along with the HFD for 3 weeks. They assessed the influence of BPA exposure on oxygen consumption using succinate and palmitoyl-carnitine as substrates, finding a decrease in oxygen consumption compared to HFD mice not treated with BPA in both scenarios. The significant decline in hepatic mitochondrial respiratory capacity was associated with intensified oxidative stress due to BPA, as evidenced by elevated hepatic ROS production and malondialdehyde (MDA) levels, which were already heightened by the HFD alone. Furthermore, BPA hindered antioxidant defense mechanisms, leading to reduced mitochondrial activity of superoxide dismutase (SOD) and aconitase enzymes. A few years later, Lee et al. explored the impact of BPA-induced hepatic lipid accumulation on the pathology of non-alcoholic fatty liver disease (NAFLD) and its underlying mechanisms, both in vitro and in vivo [59]. In their study, BPA exposure increased intracellular ROS levels and promoted fatty acid uptake by up-regulating the expression of cluster of differentiation 36, a free fatty acid transporter, in HUH-7 cells. In the in vivo experiments, C57BL/6 mice were fed a high-fat/high-cholesterol/high-cholic acid diet along with BPA at a dose of 50 mg/kg body weight for 8 weeks. These mice developed a steatohepatitis-like condition, characterized by the presence of alpha-smooth muscle actin, an indicator of hepatic fibrosis, and cleaved caspase 3, an indicator of apoptosis, in their liver tissue. Additionally, they exhibited higher levels of 8-hydroxydeoxyguanosine, a marker of oxidative stress, in their liver tissue.

Liu et al. showed that BPA exposure caused hepatoxicity through apoptosis and the sirtuin 1/peroxisome proliferator-activated receptor-γ coactivator-1a (SIRT1/PGC-1α) pathway [60]. Male Sprague Dawley rats were administered varying doses of BPA (30, 90 and 270 mg/kg body weight) via gavage for 30 days. The findings indicated that the highest BPA dose resulted in a reduction of SOD and glutathione levels, with increasing MDA levels. Additionally, rats exposed to high doses of BPA exhibited significant increases in serum alanine aminotransferase (ALT), aspartate aminotransferase (AST), total cholesterol and low-density lipoprotein cholesterol, along with a significant decrease in high-density lipoprotein cholesterol. Gene expression analysis revealed decreased levels of PGC-1α and nuclear respiratory factor 1 (Nrf1) in the liver, along with reduced protein levels of SIRT1, PGC-1α, nuclear factor erythroid 2-related factor 2 (Nrf2) and mitochondrial transcription factor A (TFAM). Conversely, the protein expression of interleukin (IL)-1β was significantly elevated in BPA-treated rats. Moreover, BPA exposure impaired mitochondrial function in hepatocytes and induced cell apoptosis in the liver, as evidenced by the up-regulation of B-cell lymphoma-2 (Bcl-2)-associated X (Bax), cleaved-caspase3 and cleaved-poly [ADP-ribose] polymerase 1 (PARP1) proteins and the down-regulation of Bcl-2.

Due to emerging literature suggesting that BPA may increase the risk of metabolic syndrome when exposure occurs in early life, Jiang et al. investigated this hypothesis on liver tissues of rat offspring at postnatal 3, 15 and 26 weeks [61]. They aimed to demonstrate that perinatal exposure to BPA predisposed offspring to NAFLD and the hepatic manifestation of metabolic syndrome and to elucidate its pathogenesis. Pregnant Wistar rats were administered BPA (40 µg/kg/day) or vehicle during gestation and lactation. At 3 weeks of age, the abnormality found in offspring was a decrease in the activity of complexes I and III of the ETS, along with significant changes in gene expression involved in mitochondrial fatty acid metabolism. At 15 weeks, they observed micro-vesicular steatosis in the liver, up-regulated genes involved in lipogenesis pathways, increased ROS generation and cytochrome c release. Furthermore, at 26 weeks, they reported extensive fatty accumulation in the liver and elevated serum alanine transaminase. During the long-term study, the hepatic mitochondrial function, including the activity of respiratory complexes, ATP synthesis, ROS generation and MMP, progressively deteriorated.

Prenatal exposure to BPA is particularly critical as it can disrupt tissue development and increase the risk of disease in adulthood. Linillos-Pradillo et al. aimed to assess whether administering BPA (0.036 mg/kg bw/day and 3.42 mg/kg bw/day) to pregnant rats would induce liver injury through oxidative stress, inflammation and apoptosis and if these effects would be evident in female offspring at postnatal day 6 (PND6) [62]. The results showed that even a low dose of BPA caused liver damage in lactating dams and had perinatal effects on female PND6 offspring. This was evidenced by increased oxidative stress levels, an inflammatory response and activation of apoptosis pathways in the liver, the organ responsible for detoxifying this endocrine disruptor.

Qiu et al. examined a number of typical immune-related parameters and oxidative stress indices in the liver of the red common carp (*Cyprinus carpio*) following exposure to five different concentrations (0.1, 1, 10, 100 and 1000 μg/L) of BPA [63]. They reported the concentration-dependent suppression of the antioxidant defense (inhibition of catalase, superoxide dismutase and glutathione peroxidase) and increased lipid peroxidation as assessed by the MDA content. Interestingly, these authors also reported BPA-related immunotoxicity since the lowest doses (0.1 and 1 μg/L) elicited an increase in immunoglobulin M and the C3 component of the complement system in both fish liver and serum.

More recently, Afzal et al. aimed to investigate the toxic effects of BPA on hematology, serum biochemistry and histopathology of different organs of common carp (*Cyprinus carpio*) [64]. When fish were exposed to BPA concentrations of 4.5 and 6 mg/L, all the organs (brain, liver, gills and kidneys) exhibited significantly lower values of the antioxidant enzymes, while fish subjected to 3 mg/L of BPA showed no significant changes.

In a recent meta-analysis examining the connection between BPA exposure and oxidative damage in rodents, Zhang et al. presented compelling evidence demonstrating that BPA exposure has a notable effect on inducing oxidative damage in these animals [65]. The study revealed that BPA significantly raised MDA levels while concurrently reducing antioxidant levels, such as glutathione reductase (GR), catalase (CAT), glutathione-S-transferase (GST), reduced glutathione (GSH) and SOD in rats/mice, with the degree of oxidative damage linked to BPA dosage, target tissue, intervention methods and exposure duration.

Ijaz et al. tested tangeretin, a natural flavonoid with various pharmacological benefits, for its protective effects against BPA-induced liver damage in male albino rats [66]. BPA exposure significantly reduced the activities of CAT, SOD, peroxidase, GR, GST and GSH levels while it increased thiobarbituric acid-reactive substances and hydrogen peroxide levels. BPA also raised levels of ALT, ALP, AST and inflammatory markers such as tumor necrosis factor-a (TNF-a), nuclear factor kappa-B (NF-κB), IL-6, IL-1β and cyclooxygenase-2 and caused histopathological damage. Co-treatment with tangeretin significantly reduced these biochemical, inflammatory and histopathological changes. This study indicates that tangeretin can effectively prevent BPA-induced liver damage due to its antioxidant and anti-inflammatory properties.

In a novel, interesting investigation, Nagarajan et al. delved into the detrimental impacts of a low dose of BPA (50 μg/kg) on enzymatic and molecular factors within hepatic tissue amidst a hypertensive environment established in male Wistar rats over a 30-day experimental period [67]. The findings reveal that exposure to BPA exacerbates the tissue abnormalities associated with hypertension, such as hepatic fibrosis, oxidative stress, elevated ACE activity, impairment of the antioxidant system, lipid irregularities and increased expression of inflammatory factors like TNF-α and IL-6. Additionally, in cellular studies, BPA was found to heighten the generation of ROS, induce mitochondrial dysfunction and escalate lipid peroxidation.

BPA substitutes, such as bisphenol S (BPS), bisphenol F (BPF) and bisphenol AF (BPAF), have received considerable attention in the search for a safer alternative. Thus, researchers have explored the impact of these BPA analogs on mitochondrial function and structure. Meng et al. explored the effects of perinatal exposure to BPA, BPF and BPAF on liver function in male mouse offspring [68]. While exposure to BPA and BPF resulted in alterations involving oxidative damage that may contribute to metabolic disorders, the BPAF treatment group did not present altered parameters. However, of all the bisphenol analogs, BPS has been mostly studied since it is the most commonly used substitute for BPA. BPS is used in the manufacture of synthetic fibers and rigid plastics. BPA or BPS exposure may alter the expression of proteins necessary for maintaining key mitochondrial respiratory processes, indicating disruption of normal mitochondrial function in the liver [69]. These compounds also impair the expression of antioxidant enzymes, indicating an increase in oxidative stress in the liver [69,70].

Growing public health concerns suggest that substantial bisphenol-mediated effects may impact liver function, especially in newborns exposed to BPA and BPS postnatally. However, the acute postnatal effects of BPA and BPS and their molecular mechanisms affecting liver function remain unclear. Liu et al. investigated the acute postnatal effects of BPA and BPS on biomarkers of liver function, including oxidative stress, inflammation, apoptosis and mitochondrial activity, in male Long–Evans rats [71]. BPA and BPS (5 and 20 μg/L of drinking water) were administered to 21-day-old male rats for 14 days. BPS showed no significant effects on apoptosis, inflammation, or mitochondrial function, but it significantly reduced ROS and nitrite content, indicating hepatoprotective effects. As expected from current scientific literature, BPA induced significant hepatotoxicity, evidenced by a substantial depletion of glutathione.

Mahim et al. demonstrated that BPS acts as a pollutant with oxidative potential by disrupting antioxidant enzymes [72]. Their study examined the impact of this BPA substitute on *Labeo rohita*, a freshwater fish, revealing that sublethal BPS exposure significantly affected the activities of hepatic antioxidant enzymes and the nonenzymatic antioxidant glutathione. Additionally, exposure altered lipid peroxidation (LPO) products, including MDA and conjugated diene levels. These changes in antioxidant levels and LPO products indicate that the fish underwent oxidative stress due to BPS exposure.

Several epidemiological studies that investigated the association between the urinary concentrations of BPA analogs (BPS and/or BPF) and the incidence of obesity/diabetes reported that BPS and BPF were detected in urinary samples at median concentrations of 0.03 to 0.4 µg/L. A BPS value of 0.4 µg/L in urine was associated with the development of obesity. Paradoxically, BPS elicited worse obesogenic effects than BPA, which were related to the induction of oxidative stress, inflammation and the impaired gene expression of adipogenesis-related markers. Alarmingly enough, the lower BPS urinary concentrations (0.1–0.03 µg/L) were found to be associated with diabetes by some authors [73]. 

Interestingly, a recent in vitro study carried out in a human adrenocortical carcinoma H295R cell line reported biphasic, hormetic effects of bisphenols S, F, B and AF in biological systems since cytotoxicity was elicited only by the high doses, while low doses improved both cellular viability and steroid hormone secretion [74].

### 4.2. Alkylphenols

Along with BPA, nonylphenol (NP) is a widely distributed endocrine-disrupting compound. NP is a persistent and highly toxic EDC with estrogenic properties that has harmful effects on both humans and wildlife. Shi et al. estimated the combined toxicity of BPA and NP at a clinically safe dose in rats [75]. As anticipated, oxidative stress was involved in BPA- and NP-induced toxicity, with elevated levels of MDA and decreased activity of antioxidant enzymes. However, the combined exposure seemed to have an unexpectedly reduced toxicity as compared to either the BPA or NP group. Mukherjee et al. have used zebrafish to investigate the impact of NP on hepatic redox homeostasis [76]. Their results were similar to those described in the study by Shi et al., namely that chronic exposure to NP promoted oxidative stress.

### 4.3. Polychlorinated Biphenyls (PCBs)

PCBs are synthetic organochlorine chemicals, categorized as “probably carcinogenic to humans” that belong to the group of persistent organic pollutants due to their resistance to environmental degradation, which can bioaccumulate due to their chemical and thermal stability and magnify along the food chains [77]. Besides the carcinogenic effects, PCBs and their mixtures have been reported to elicit neurotoxicity, immunotoxicity and cardiovascular and reproductive toxicity by increasing oxidative stress and impairing various metabolic pathways [78,79]. Their in vivo metabolic transformation results in hydroxylated PCBs (OH-PCBs), which have been detected in human serum and are responsible for several deleterious effects, e.g., the formation of covalent adducts with DNA and other macromolecules, inhibition of enzymes that regulate hormone concentrations and interference with hormone transport and signaling [80].

Van Etten et al. demonstrated that exposure to PCBs and 2,3,7,8-tetrachlorodibenzo-p-dioxin (TCDD) resulted in mitochondrial damage and changes in mtDNA copy number since exposure to these substances has been demonstrated to enhance oxidative stress [81]. TCDD is one of a family of isomers known chemically as dibenzo-p-dioxins, employed as a research chemical even though it has no recognized commercial applications. It was tested as a pesticide against insects and fungi that degrade wood, but it was never utilized commercially. This study was designed to determine the connection between exposures to TCDD and PCBs and mtDNA copy number in a unique set of rat liver tissue samples obtained as a result of earlier studies conducted by the National Toxicology Program (NTP) [81]. This research provides the information required to determine whether mitochondria are the targets of TCDD and PCB toxicity and if mtDNA copy number may be a molecular biomarker associated with the toxicity of these persistent environmental pollutants [81]. The doses used during the NTP studies were based on World Health Organization TEF recommendations for toxicological equivalence [82], while the doses of the binary mixture of PCB 126 and PCB 153 were chosen to reflect the environmentally relevant 1:1000 ratio of these analogs [83]. The results show an increase in mtDNA copy number, which may be used as a sensitive biomarker for the early detection of mitochondrial injury and oxidative stress [81].

Ounnas et al. investigated the in vivo toxicity of PCB using a realistic exposure model, wherein rats were chronically administered a low daily dose of PCBs (twice the TDI) for 8 weeks. The study focused on assessing PCB-induced toxic effects on the liver and brain, specifically examining oxidative stress levels and mitochondrial function. The results indicated that even at low doses, chronic exposure to PCBs led to a significant decrease in mitochondrial function in both liver and brain tissues. In the liver, oxygen consumption during ATP production (state 3) decreased. Additionally, PCB toxicity in the liver was assessed through transaminase enzymatic activity, revealing elevated lipid peroxidation status and transaminase activity in the blood of exposed rats [84]. A few years later, Deng et al. aimed to understand the intricate effects of PCB 126, a toxic compound, on liver metabolism, particularly in the context of liver injury [85]. Utilizing metabolomics techniques, researchers compared the liver metabolites affected by PCB 126 in control mice versus those in a mouse model with diet-induced liver injury. Over 14 weeks, mice were fed either a standard diet or diet-inducing non-alcoholic steatohepatitis before PCB 126 exposure. The results revealed distinct differences in hepatic metabolomics profiles between PCB 126-exposed and control mice, with significant alterations in various metabolic pathways, including glycerophospholipid metabolism, glutathione metabolism, and CoA biosynthesis. Interestingly, these effects were consistent regardless of diet, indicating general markers of PCB 126 exposure. Furthermore, PCB 126 exposure led to elevated levels of metabolites associated with oxidative stress and mitochondrial dysfunction, particularly pronounced in mice with compromised livers. Notably, PCB 126 downregulated redox genes, with a more significant impact observed in mice with liver injury. Overall, the study highlights PCB 126’s ability to induce oxidative stress and metabolic dysfunction, with pre-existing liver injury exacerbating these effects. The findings underscore the importance of considering liver health in assessing PCB 126 toxicity, elucidating potential mechanisms of enhanced toxicity in liver injury conditions through metabolic profiling.

### 4.4. Triazines

Atrazine (ATZ) is a widely used herbicide that belongs to the triazine class of herbicides, which are characterized by their selective action on broadleaf and grassy weeds. The primary sources of atrazine contamination are agricultural runoff, which carries the chemical into surface- and groundwater, posing potential risks to ecosystems and human health. Its prevalence and persistence in the environment have made atrazine a focal point in discussions of agricultural practices and environmental protection. ATZ has been linked to metabolic diseases, but little is known about how it affects mitochondrial function. Therefore, Jin et al. studied the effects of ATZ and its metabolite diaminochlorotriazine (DACT) on the induction of oxidative stress and endocrine disruption [86]. Both the low and high doses of DACT, as well as the high dose of ATZ, presented a significant increase in SOD activity in the liver [86]. However, CAT activity in the liver significantly diminished only in the group receiving a high dose of DACT [86]. Two years later, Sagarkar et al. examined how short-term ATZ exposure affected the expression of a number of nuclear and mitochondrial-encoded genes involved in oxidative phosphorylation in HepG2 cells [87]. Mitochondrial toxicity was indicated by a reduction in ATP content following ATZ exposure with an EC50 value of 0.162 mM for ATZ-related mitotoxicity [87].

### 4.5. Phthalates

Phthalates are plasticizers that represent the majority of soft polyvinyl chloride plastics [88,89,90]. The most widely produced phthalate ester is di-(2-ethylhexyl) phthalate (DEHP), frequently added to industrial plastics, food packaging, cosmetics, kids’ toys and medical equipment like hemodialysis tubes and blood storage bags [90,91,92]. Through thorough research, it has been shown that DEHP results in hepatotoxicity. However, the pathogenesis of DEHP-induced hepatic injury is still under investigation. To generate long-term, low-dose DEHP exposure, Lee et al. subjected hepatic stellate cells (HSC)—T6 rat hepatic cell line—to 50 and 100 µM DEHP for 3.5 months [93]. They investigated the effect of chronic DEHP exposure on mitochondrial respiration in HSCs and concluded that long-term exposure to low-dose DEHP induces mitochondrial dysfunction and may influence apoptosis in HSCs, as DEHP-exposed HSCs had a decreased capacity to produce ATP, impaired mitochondrial maximal working capacity and a lower level of both spare respiratory capacity and basal oxygen consumption [93]. In order to further describe the hepatic responses to long-term DEHP exposure, Li et al. performed extensive metabolomics and transcriptomics analyses and systematically analyzed the pathogenesis and mechanisms of liver damage [94]. Consistent with a previous study conducted by Zhang et al., it was determined that DEHP exposure increased the level of MDA and the peroxidation product and decreased antioxidant function [94,95]. Mono(2-ethylhexyl) phthalate (MEHP) is the main DEHP metabolite and the product of DEHP conversion by lipids. Due to its known toxicity, Park et al. examined the antioxidant and oxidative stress changes in zebrafish liver cells [96]. Interestingly, even though MEHP-treated cells had higher levels of ROS, they had lower levels of LPO.

### 4.6. Per- and Polyfluoroalkyl Substances (PFASs)

Perfluorooctane sulfonate (PFOS), also known as perfluorooctanesulfonic acid, is a manufactured global pollutant, that, according to research, can cause liver damage by accumulation [97]. Seeing as the molecular mechanism of PFOS-induced hepatotoxicity remains unclear, Han et al. investigated whether PFOS-induced oxidative stress contributes significantly to liver injury [97]. In rats treated with PFOS, there was a dose-dependent increase in ROS production. Moreover, similar to the previous studies described, the estimation of MDA concentration was used to determine the final product of lipid peroxidation, and it was found to be significantly increased [97]. Additionally, PFOS considerably lowered the activity of antioxidant enzymes, as was expected.

### 4.7. Polychlorinated Diphenyl Ethers (PCDEs)

These compounds have been discovered in a variety of environmental samples, and their ubiquitous distribution is mostly due to their existence as impurities in chlorophenol preparations [98]. A recent study provided more knowledge on the harmful effects of PCDEs in fish, along with additional toxicological data on PCDEs on aquatic species [99]. After in vivo PCDE exposure, both liver antioxidant enzyme activities and MDA concentrations changed in a dose-dependent manner [99]. In zebrafish tissues, 4-mono-CDE and 4,4′-di-CDE caused the most severe oxidative stress of the five PCDE congeners studied [99]. Through their research, Ye et al. contribute to the evaluation of the risk posed by PCDEs to aquatic ecosystems [99].

### 4.8. Mixtures of EDCs

Due to the current lifestyle, the possible health concerns associated with regular exposure to low doses of EDC mixtures are a major issue [100]. Thus, the research on the cumulative effects of multiple EDCs has major relevance for public health, mainly because exposure to these environmental pollutants does not occur singularly. On a regular basis, people are exposed to mixtures of endocrine-disrupting compounds at concentrations close to, or much below, the existing regulatory limits [101]. Thus, the approach known as real-life risk simulation or real-life exposure scenario evaluates the biological or clinical effects of chemical combinations given over a long period of time at low doses [102]. Consequently, Vardakas et al. studied the effects of a mixture containing endocrine-disrupting chemicals (the chemicals methylparaben (MePB), butylparaben (BuPB), propylparaben (PrPB) and triclosan (TCS), which are preservatives or antimicrobial agents, and the synthetic compounds BPA and DEHP) and a herbicide called Roundup^®^, along with its active ingredient, glyphosate, administered in a long-term, low-dose manner [101]. A parameter worth taking into account is the fact that the animals in this study (rabbits) were exposed to the xenobiotics for only 12 months, a length significantly shorter than their normal life expectancy [101]. Oxidative stress was induced by the exposure to both EDC mixtures and Roundup^®^, whereas glyphosate did not affect it [101]. This indicates that the long-term, low-dose regimen used in this study had a detrimental impact on the liver’s redox status, a vital organ involved in the biological process of detoxifying organisms from xenobiotics [101].

Table 1 summarizes the EDC-related liver toxic effects with respect to mitochondrial dysfunction and oxidative stress, according to time exposure (acute ≤ 2 days, chronic > 2 days).

**Table 1:** EDCs and liver toxicity in experimental models.

Endocrine Disruptor	Experimental Model	Dose	Duration	Effects	Ref.
Bisphenol A (BPA)	HepG2 cells	$10^{-4}$ to $10^{-12}$ M	Acute: 24, 48 (and 72) h	₋ Increased ROS production ₋ Hyperpolarization of MMP	[50]
Bisphenol A (BPA)	HepG2 cells	10 or 100 nM	Acute: 2, 6, 12 or 24 h	₋ Decreased OCR and ATP production ₋ Decreased MMP ₋ Increased MDA concentrations	[51]
	Mouse liver	1.2 mg/kg bw/day	Chronic: 5 days	₋ Swollen mitochondria ₋ Decreased OCR ₋ Decreased expression of respiratory complexes III and V ₋ Increased levels of MDA ₋ Decreased expression of GPx3	
Bisphenol A (BPA)	Mouse liver	BPA water 50 μg/kg/day	Chronic: 10 weeks	₋ Increased ROS, RNS, MDA ₋ Decreased activities of SOD, GPx, CAT and T-AOC ₋ Decreased activity of ETS complexes I–V ₋ Decreased intracellular ATP content	[56]
Bisphenol A (BPA)	Mouse liver	50 µg/kg along with HFD (after 12 weeks of HFD feeding)	Chronic: 3 weeks	₋ Decreased state 3 respiration ₋ Increased ROS production ₋ Increased MDA levels ₋ Decreased mitochondrial activity of SOD and aconitase enzymes	[58]
Bisphenol A	Mouse liver	50 mg/kg/bw along with HFCCD bw	Chronic: 8 weeks	₋ Increased α-SMA and cleaved caspase 3 ₋ Increased index of 8-OHdG	[59]
Bisphenol A (BPA)	Neonatal rat liver	1.47 ng/mL	Acute: 1 h	₋ Swollen mitochondria	[103]
Bisphenol A (BPA)	Rat liver	30 mg/kg bw/day	Chronic: 6 weeks	₋ Decreased activities of GSHPx, GR, SOD and GSH ₋ Increased level of MDA	[57]
Bisphenol A (BPA)	Rat liver	50 mg/kg/day	Chronic: 4 weeks	₋ Decreased levels of GSH and SOD ₋ Decreased activities of GSHPx, CAT, GR and GST ₋ Increased TBARS and NO	[52]
Bisphenol A (BPA)	Rat liver	150 mg/kg, 250 mg/kg and 500 mg/kg	Chronic: 14 days	₋ Decreased activity of complexes I–V of ETS ₋ Decreased GSH level ₋ Increased LPO level ₋ Increased protein carbonyl content ₋ Increased production of superoxide anion ₋ Decreased activity of GPx, GR and SOD dismutase	[53]
Bisphenol A (BPA)	Rat liver	10 and 50 mg/kg	Chronic: 30 days	₋ Increased levels of MDA (at 50 mg/kg dosage) and H_2_O_2_ (at 10 and 50 mg/kg dosage) ₋ Decreased levels of CAT, GSHPx and SOD (at 50 mg/kg dosage)	[54]
Bisphenol A (BPA)	Rat liver	0.5 mg/kg (low dose), 5 mg/kg (medium dose, NOAEL) or 50 mg/kg (high dose)	Chronic: 30 days	₋ Increased levels of MDA in all treated groups ₋ Decreased GSH content in all treated groups	[55]
Bisphenol A (BPA)	Rat liver	100 mg/kg	Chronic: 30 days	₋ Decreased activities of CAT, SOD, POD, GSR, GST and GSH ₋ Increased TBARS and H_2_O_2_ levels ₋ Increased TNF-α, NF-κB, IL-6, IL-1β levels and COX-2 activity	[66]
Bisphenol A (BPA)	Rat liver	90 and 270 mg/kg bw	Chronic: 30 days	₋ Decreased SOD and GSH levels (high dose) ₋ Increased MDA level (high dose) ₋ Decreased gene expression of PGC-1α and Nrf1 (high dose) ₋ Decreased protein levels of SIRT1, PGC-1α, Nrf2 and TFAM (high dose) ₋ Increased protein expression of IL-1β ₋ Increased cell apoptosis by up-regulating the protein levels of Bax, cleaved-Caspase3 and cleaved-PARP1 and down-regulating the Bcl-2	[60]
Bisphenol A (BPA)	Rat liver (L-NAME-induced hypertensive Wistar rats)	50 µg/kg	Chronic: 30 days	₋ Potentiated oxidative stress, ACE activity, malfunction of the antioxidant system, lipid abnormalities and inflammatory factor (TNF-α and IL-6) expression	[67]
Bisphenol A (BPA)	Rat offspring liver	40 µg/kg/day	Chronic: during gestation and lactation	₋ At 3 weeks of age: Decreased MRC activity (I and III) ₋ At 15 weeks: Restricted activities of CI, CII and CIII Decreased ATP production Increased ROS generation ₋ At 26 weeks: Decreased all MRC activities Decreased MMP Decreased ATP production Increased ROS generation	[61]
Bisphenol A	Rat liver (from pregnant rats and female postnatal day-6 offspring)	0.036 mg/kg bw/day and 3.42 mg/kg bw/day	Chronic: during premating, mating, pregnancy, lactation	₋ Increased oxidative stress levels ₋ Increased inflammatory response ₋ Increased apoptosis pathways	[62]
Bisphenol A (BPA)	Common carp (*Cyprinus carpio*) liver	0.1, 1, 10, 100 and 1000 µg/L	Chronic: 30 days	₋ Inhibited CAT activity (at concentrations of 100 and 1000 µg/L) ₋ Decreased GSH-Px and SOD activities (at concentrations of 10, 100 and 1000 µg/L) ₋ Increased LPO (assessed by MDA content) with the increase in BPA concentration (at concentrations of 100 and 1000 µg/L)	[63]
Bisphenol A (BPA)	Common carp (*Cyprinus carpio*) liver	4.5 and 6 mg/L	Chronic: 30 days	₋ Increased levels of ROS and TBARS ₋ Decreased levels of SOD, CAT POD	[64]
Bisphenol A (BPA) and S (BPS)	Rat liver	50 or 500 μg/kg/day of BPA or BPS; 50 μg/kg/day of both BPA and BPS	Chronic: 20 weeks	₋ Up-regulated the expression of Sdhb (exposure to BPA or BPS) ₋ Up-regulated the expression of COQ9 and COX5B (exposure to BPS) ₋ Dysregulated expression of liver antioxidant enzymes	[69]
Bisphenol S (BPS)	Mouse liver	100 µg/kg/day in drinking water	Chronic: 10 weeks	₋ Disrupted mitochondrial function and oxidative stress parameters	[104]
Bisphenol S (BPS)	Mouse liver	0.1–1 mM	Acute: 12 h	₋ Increased ROS	[70]
Bisphenol S (BPS)	Fish (*Labeo rohita*) liver	Groups II, III and IV were exposed to 80 mg BPS/L, Groups V, VI and VII were exposed to 100 mg BPS/L, and Groups VIII, IX and X to 120 mg BPS/L	Chronic: 7, 14 and 21 days	₋ Increased activities of SOD, CAT, GR and GSH ₋ Decreased activities of GPx and GST	[72]
Bisphenols: bisphenol A (BPA), bisphenol F (BPF) and bisphenol AF (BPAF)	Mouse offspring liver	100 ng/g bw/day	Chronic: from the 7th day of pregnancy to the 21st day after delivery	₋ BPA: Decreased activities of SOD, CAT and GSH-Px and level of GSH Increased level of MDA ₋ BPF: Decreased activities of CAT and GSH	[68]
Bisphenol A (BPA) + nonylphenol (NP)	Rat liver	100 µg/kg	Chronic: 56 consecutive days	₋ Increased MDA ₋ Decreased activities of SOD, GSH, GSH-Px, GSH-ST, CAT and POD	[75]
Nonylphenol (NP)	Zebrafish (*Danio rerio*) liver	50 and 100 μg/L	Chronic: 21 days	₋ Promotes ROS synthesis, more specifically, superoxide anions and H_2_O_2_ levels	[76]
Polychlorinated biphenyls (PCBs)	Rat liver	Contaminated (two times the TDI) goat milk administered daily by gavage (6 µL/g bw)	Chronic: 8 weeks	₋ Decreased oxygen consumption and ATP production ₋ Increased TBARS	[84]
Polychlorinated biphenyls (PCBs)—PCB126	Mouse liver	1.53 μmol/kg	Chronic: 10 weeks	₋ Decreased level of GSH	[85]
Polychlorinated biphenyls (PCBs) and 2,3,7,8-Tetrachlorodibenzo-p-dioxin (TCDD)	Rat liver	TCDD at 3, 10 or 100 ng/kg/day; PCB 126 at 10, 100 or 1000 ng/kg/day; PCB 153 at 10, 100 or 1000 μg/kg/day; Binary mixture of PCB 126 and PCB 153 (10 ng/kg/day + 10 μg/kg/day, 100 ng/kg/day + 100 μg/kg/day or 1000 ng/kg/day + 1000 μg/kg/day	Chronic: 13 and 52 weeks	₋ Increased mtDNA copy number (TCDD exposure and mixture of PCB126 and PCB153 exposure)	[81]
Atrazine (ATZ) and its metabolite diaminochlorotriazine (DACT)	Mouse liver	ATZ and DACT (100 and 200 mg/kg/bw, respectively)	Chronic: 1 week	₋ Increased SOD activities (high dose of ATZ treated group and both of the low and high doses of DACT treated groups) ₋ Decreased CAT activity (high dose of DACT treated group) ₋ Increased GST activity (high dose of DACT treated group)	[86]
Atrazine (ATZ)	HepG2 cells	0.05–2 mM	Acute: 3 h and 6 h	₋ Decreased ATP content ₋ Down-regulation of many OXPHOS subunit expression ₋ Up-regulated SOD and SIRT3 expression ₋ Down-regulated TFAM and SIRT1 expression	[87]
Di (2-ethylhexyl) phthalate (DEHP)	HSC-T6 cells	50 and 100 µM	Chronic: 3.5 months	₋ Decreased ability to produce ATP ₋ Impaired mitochondrial maximal working capacity ₋ Decreased level of spare respiratory capacity and basal oxygen consumption	[93]
Di (2-ethylhexyl) phthalate (DEHP)	Rat liver	600 mg/kg/day	Chronic: 12 weeks	₋ Increased level of MDA ₋ Decreased levels of SOD, CAT, GSH-Px, T-AOC and GSH/GSSG	[94]
Di (2-ethylhexyl) phthalate (DEHP)	Female quail (*Coturnix japonica*) liver	250, 500 and 1000 mg/kg bw/day	Chronic: 45 days	₋ Increased MDA, GSH and GST ₋ Decreased T-AOC, SOD and GPx ₋ Induced mitochondrial ultrastructural abnormalities and mitochondrial dysfunctions ₋ Activated mitochondrial unfolded protein response	[95]
Mono-(2-ethylhexyl) phthalate (MEHP)	Zebrafish liver	31.25, 62.5, 125, 250, 500 or 1000 mg/L	Acute: 24 h	₋ Increased GST and GPx activities ₋ Decreased MDA level ₋ Increased SOD activity (125 mg/L) ₋ Increased total GSH concentration (62.5 mg/L)	[96]
Perfluorooctane sulphonate (PFOS)	Rat liver	Single dose of 1 or 10 mg/kg body	Chronic: 28 consecutive days	₋ Increased intracellular ROS and NO production ₋ Weakened intracellular antioxidant defense by inhibiting CAT and SOD activities ₋ Elevated iNOS, Bax, cytochrome c, cleaved caspase-9 and cleaved caspase-3	[97]
Polychlorinated diphenyl ethers (PCDEs)	Zebrafish liver	1, 10 and 50 μg/L	Chronic: 14 days	₋ Induced oxidative stress	[99]
Endocrine disruptors mixture	Rabbit liver	10 × ADI	Chronic: 12 months	₋ Decreased CAT activity ₋ Increased TBARS levels	[101]

8-OHdG = 8-hydroxydeoxyguanosine; α-SMA = alpha-smooth muscle actin; ACE = angiotensin converting enzyme; ADI = acceptable daily intake; ATP = adenosine triphosphate; Bax = Bcl-2-associated X; Bcl-2 = B-cell lymphoma-2; bw = body weight; CAT = catalase; COQ9 = ubiquinone biosynthesis protein COQ9_ mitochondrial; COX-2 = cyclooxygenase-2; COX5B = cytochrome c oxidase subunit 5B mitochondrial; DACT = diaminochlorotriazine; ETS = electron transport system; GPx3 = glutathione peroxidase 3; GR = glutathione reductase; GSH = glutathione; GSHPx = glutathione peroxidase; GST = glutathione-S-transferase; GSSG = glutathione disulfide; HFCCD = high-fat/high-cholesterol/high-cholic acid diet; HFD = high-fat diet; HSC = hepatic stellate cells; IL = interleukin; iNOS = inducible nitric oxide synthase; Nrf = nuclear respiratory factor; L-NAME = Nω-nitro-l-arginine methyl ester; LPO = lipid peroxidation; MDA = malondialdehyde; MMP = mitochondrial membrane potential; MRC = mitochondrial respiratory chain; mtDNA = mitochondrial DNA; NF-κB = nuclear factor kappa-B; NO = nitric oxide; NOAEL = no adverse effect level; OCR = oxygen consumption rate; OXPHOS = oxidative phosphorylation; PARP-1 = poly [ADP-ribose] polymerase 1; PGC-1α = peroxisome proliferator-activated receptor-gamma coactivator 1α; POD = peroxidase; ROS = reactive oxygen species; RNS = reactive nitrogen species; SIRT1 = sirtuin 1; SIRT3 = sirtuin 3; SDHB = succinate dehydrogenase iron-sulfur subunit; SOD = superoxide dismutase; T-AOC = total antioxidant capacity; TBARS = thiobarbituric acid reactive substances; TDI = tolerable daily intake; TFAM = mitochondrial transcription factor A; TNF-α = tumor necrosis factor-α.

## 5. EDCs and Pancreatic Toxicity

The endocrine pancreas consists of the islets of Langerhans, a diverse population of 1000–3000 cells, where the most common cell type is the insulin-releasing β-cell [13].

A growing number of studies suggest a link between exposure to EDCs and the development of several pancreatic diseases, including diabetes, pancreatitis and pancreatic cancer [105].

### 5.1. Bisphenols (BPs)

Wei et al. evaluated how early-life BPA exposure affects metabolic syndrome in rat offspring fed a normal diet and a high-fat diet [106]. They mostly found alterations of morphologic features, such as swollen mitochondria. BPA was also demonstrated to cause damage to mitochondrial function and metabolism in cultures of primary murine pancreatic islets under low-dose treatment [107]. This conclusion was reached after a time-dependent increase in intracellular ROS level after 12 h exposure, as well as a further decrease of MMP at 18 and 24 h [107]. Research conducted by Susiarjo et al. has established that two generations of adult male mice offspring exposed via maternal transmission to relevant human exposure levels of BPA prior to conception and throughout lactation presented with increased body fat, impaired glucose tolerance and decreased glucose-stimulated insulin secretion (GSIS) [108]. Therefore, Bansal et al. have aimed to discover the multigenerational effect of maternal BPA exposure on mouse pancreatic islets [109]. In order to establish whether impaired GSIS in two generations of adult offspring is due to altered mitochondrial-driven insulin secretion, they evaluated insulin secretion in response to α-ketoisocaproate, a substrate processed in mitochondria. Interestingly, only the male offspring receiving the higher dose of BPA presented an abnormal mitochondrial phenotype, as opposed to male offspring exposed to the low dose of BPA and female offspring, regardless of the dosage [109]. As a result, all subsequent research was limited to adult male offspring. Furthermore, they performed high-resolution respirometry to quantify oxygen consumption in intact islet mitochondria and investigate whether the phenotype in males was linked to mitochondrial dysfunction. Accordingly, they found reduced basal and maximal oxygen consumption in the intact islets of the two generations of male offspring under the higher dose of BPA. Interestingly, opposite to the results of the previous studies described, one study led by Moon et al. found no significant differences in the morphology or number of islet cells or mitochondria in the pancreas of mice under long-term exposure to BPA [110].

### 5.2. Alkylphenols

Phenolic estrogen pollutants have captured the attention of researchers, largely because of their estrogenic activities that mimic the actions of the steroid hormone 17b-estradiol [111]. Revolving around the hypothesis that exposure to these contaminants may impair insulin secretion and increase the risk of type 2 diabetes, Song et al. investigated the direct effects of phenolic estrogen diethylstilbestrol (DES), octylphenol (OP), NP and BPA on rat pancreatic islets in vitro, whose estrogenic activities were DES > NP > OP > BPA [111]. The mitochondria in β-cells treated with NP, OP or BPA were noticeably enlarged, with higher average area and optical density, and the majority of them had structural integrity loss with abnormal cristae. Moreover, they found the pancreatic complex IV mitochondrial respiratory chain enzyme (COX) activity (that indicates mitochondrial function) was considerably lower in islets treated with NP, OP or BPA, along with lower cytosolic ATP levels. In a more recent study centered solely on the organic alkylphenol 4-NP and its adverse effects on pancreas, Sprague–Dawley rats were treated with different doses of NP for 90 consecutive days [112]. In the group treated with higher concentrations of NP, ROS production was found to be extremely increased. During apoptosis, the MMP collapses simultaneously with the opening of the mitochondrial permeability transition pores, resulting in the release of cytochrome c into the cytosol and the activation of additional downstream processes in the apoptotic cascade [112]. As a result, MMP levels in the islets were examined and discovered to be dose-dependently decreased.

### 5.3. Organotins

Tributyltin (TBT), a toxic chemical belonging to the organotin class, is the primary active component in several biocides, regarded as moderately to extremely persistent organic pollutants that pose a particular threat to marine ecosystems [113]. Chen et al. explored how noncytotoxic doses of TBT, relevant to human exposure, affect β-cell function in vitro and in vivo [114]. The β-cell-derived RIN-m5F cells and pancreatic islets from mice and humans were treated with TBT (0.05–0.2 μM) for 0.5–4 h, while adult male mice were orally exposed to TBT (25 μg/kg/day) with or without antioxidant N-acetylcysteine (NAC) for 1–3 weeks. Low concentrations of TBT were found to boost glucose-stimulated insulin secretion and intracellular calcium levels in β-cells, alongside an increased production of ROS and phosphorylation of protein kinase C (PKC) and extracellular signal-regulated kinase (ERK)1/2. These effects were reversible with antiestrogen ICI182780 and inhibitors of ROS, calcium and PKC, but not ERK. Islets treated with TBT also showed increased insulin secretion, which could be reversed by ICI182780, NAC and PKC inhibitors. In mice exposed to TBT for three weeks, elevated blood glucose and plasma insulin levels were observed, leading to glucose intolerance and insulin resistance, all of which could be reversed by NAC. These results imply that low doses of TBT disrupt insulin regulation and glucose homeostasis via estrogen receptor-regulated and/or oxidative stress-related pathways.

### 5.4. Mixture of EDCs

In a more recent study, Dos Santos et al. investigated the impact of MDCs on cell survival and glucagon secretion using the mouse α-cell line TC1-9 [115]. ROS production was assessed after exposure to BPA, TBT, perfluorooctanoic acid (PFOA), triphenylphosphate (TPP), TCS and dichlorodiphenyldichloroethylene (DDE) and increased levels were found under treatment with BPA or TBT, whilst the rest of the MDCs had no effect on ROS production. In order to validate the hypothesis that environmental pollutants can directly influence β-cell function and explore the molecular mechanisms by which these contaminants can alter β-cell function, Makaji et al. designed a study using mouse beta TC-6 cells [116]. Similarly, they investigated the acute impact of six compounds: benzopyrene, BPA, PrPB, MePB, PFOA and perfluorooctyl sulfone. The most interesting finding was the lack of effects of BPA treatment on complex IV and citrate synthase activity under any of the conditions examined.

Even though there is knowledge of the connection between BPA and pancreatic diseases [105], the underlying pathogenesis still remains unknown.

Table 2 summarizes the toxic effects of EDCs on pancreatic cells at a mitochondrial level and those associated with increased oxidative stress, according to time exposure (acute ≤ 2 days, chronic > 2 days).

## 6. Discussion

Over the past 40 years, the global prevalence of obesity and related comorbidities has surged at alarming rates [117]. Among the various environmental factors contributing to the worldwide decline in metabolic health, EDCs have attracted significant attention from the scientific community [3]. The documented rise in obesity and metabolic disorders has chronically paralleled the increased production and widespread use of EDCs [6]. Epidemiological studies have increasingly underscored a close correlation between the prevalence of EDCs and the epidemic of metabolic diseases. Complementing these epidemiological findings, increasing experimental data have elucidated several mechanisms through which EDCs disrupt the hormonal environment, thereby promoting metabolic diseases along with other predisposing factors, such as high-fat diet or stress, among others [4].

Metabolic diseases frequently correlate with mitochondrial dysfunction, making it unsurprising that several EDCs have been identified as modulators of processes critical to fatty acid and glucose metabolism, as well as energy production and utilization within mitochondria [40]. The consequences of EDC exposure include decreased electron transport system activity and ATP production and lowered MMP and mitochondrial swelling. Moreover, the EDC-driven accumulation of ROS leads to increased lipid peroxidation and protein oxidation while also enhancing mtDNA susceptibility to damage. These findings underscore the intricate mechanisms by which EDCs disrupt mitochondrial function and metabolic homeostasis, potentially contributing to the pathogenesis of metabolic diseases [118]. Understanding these complex interactions is vital for developing strategies to mitigate the adverse health effects associated with environmental exposures to EDCs, thereby safeguarding metabolic health in exposed populations.

**Figure 1:**
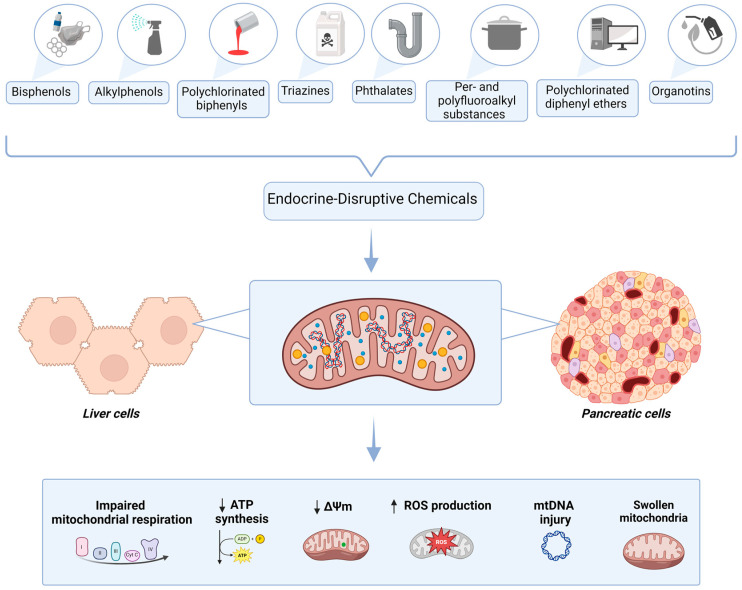
Mechanisms of liver and pancreatic EDC-related mitochondrial toxicity. Created with BioRender. Δψm = mitochondrial membrane potential; ATP = adenosine triphosphate; DNA = deoxyribonucleic acid; ROS = reactive oxygen species; ↑ = increased; ↓ = decreased.

This review has centered on elucidating the impact of EDCs on the liver and pancreas, both crucial organs to human metabolism, particularly focusing on their mitochondrial effects. This emphasis underscores mitochondrial dysfunction as a pivotal mechanism through which EDCs exert their deleterious effects on health. EDCs are recognized for their capacity to disrupt mitochondrial function, leading to compromised energy metabolism and increased oxidative stress, which are pivotal in the etiology of metabolic diseases. Disruptions in mitochondrial integrity and/or function are implicated in triggering a cascade of metabolic perturbations, including insulin resistance, adipose tissue enlargement and hepatic and pancreatic steatosis, thereby contributing significantly to metabolic dysregulation and unfavorable health outcomes. The complex mitochondrial effects of the discussed EDCs are summarized in Figure 1.

There is an unmet need to identify novel, effective testing methods for EDCs in humans in order to facilitate the detection of MDC-related contributions to metabolic diseases and to reduce exposure and, thus, disease vulnerability.

Furthermore, it is important to keep in mind that humans are exposed to a mixture of multiple EDCs, which have the potential to concurrently impact mitochondrial function and disrupt metabolic homeostasis. The complexity of these chemical mixtures poses a significant challenge in understanding their cumulative effects on human health. Studies have demonstrated that various EDCs can act synergistically or additively, amplifying their individual impacts on mitochondrial integrity and metabolic pathways [119]. This interplay between different EDCs in humans underscores the need for a research strategy aimed at elucidating the combined effects of multiple EDCs on liver, pancreas and adipose tissue, using samples harvested from patients with metabolic diseases and their complications.

New preventive strategies and legislative initiatives must consider the identification of substances that particularly contribute to the development of a specific pathology in order to lessen the detrimental effects of these endocrine disruptors on cardiometabolic health [120].

## 7. Conclusions

There is an increasing amount of research tackling the effects of EDCs on mitochondria structure and function, mainly because their contribution to the development of metabolic pathologies is far from being completely elucidated. In this review, we outlined some of the effects of EDCs on mitochondria in two primary organs involved in metabolism: the liver and pancreas. EDC-induced mitochondrial dysfunction is defined by changes in mitochondrial bioenergetics, biogenesis, morphology and dynamics and activation of the mitochondrial apoptosis pathways in association with increased mitochondria-driven oxidative stress. There is an unmet need for the identification of mitochondria-targeted protective compounds able to prevent or minimize EDC-related mitochondrial dysfunction and subsequent oxidative stress.

## Figures and Tables

**Table 2:** EDCs and pancreatic toxicity in experimental models.

Endocrine Disruptor	Experimental Model	Dose	Duration	Effects	Ref.
Bisphenol A (BPA)	β-cells of rat offspring	50, 250 or 1250 µg/kg/day	Chronic: during gestation and lactation	₋ Swollen mitochondria	[106]
Bisphenol A (BPA)	Primary murine pancreatic islets	$10^{-9}$ M	Acute: 24 or 48 h	₋ Decreased MMP ₋ Increased ROS cellular levels	[107]
Bisphenol A (BPA)	Pancreatic islets of mice offspring	10 µg/kg/d (low dose) and 10 mg/kg/d (high dose)	Chronic: during gestation and lactation	₋ Decreased basal and maximal oxygen consumption in intact islets of F1 and F2 males (high dose) ₋ Increased Ogdh expression in F2 males (low dose) ₋ Increased UCP2 mRNA levels in F1 and F2 males (high + low dose)	[109]
Bisphenol A (BPA)	αTC1-9 murine cell line	From 0.1 pM to 1 µM	Acute: 24 h	₋ Increased ROS production	[115]
Tributyltin (TBT)	αTC1-9 murine cell line	From 0.1 pM to 1 µM	Acute: 24 h	₋ Increased ROS production	[115]
Tributyltin (TBT)	β-cell-derived RIN-m5F rat cell line; pancreatic islets of mice and humans	0.05–0.2 μM	Acute: 0.5–4 h	₋ Increased ROS production	[114]
Octylphenol (OP)	Pancreatic islets of rats	25 µg/L	Acute: 24 h	₋ Swollen mitochondria with loss of distinct cristae structure within the inner membrane ₋ Decreased activity of complex IV of ETS ₋ Decreased cytosolic ATP level ₋ Disruption of mRNA expression of UCP2 and Ogdh genes	[111]
Nonylphenol (NP)					
Bisphenol A (BPA)					
4-Nonylphenol (NP)	Pancreatic islets of rats	60 mg/kg and 180 mg/kg	Chronic: 90 consecutive days	₋ Increased ROS production ₋ Decreased MMP	[112]

ATP = adenosine triphosphate; ETS = electron transport system; F1 = first-generation adult mice offspring; F2 = second-generation adult mice offspring; MMP = mitochondrial membrane potential; Ogdh = oxoglutarate dehydrogenase; ROS = reactive oxygen species; UCP2 = uncoupling protein 2.

## Data Availability

The article is a narrative review. No data were generated or analyzed.

## List of Abbreviations

8-OHdG	8-hydroxydeoxyguanosine
α-SMA	alpha-smooth muscle actin
ACE	angiotensin-converting enzyme
ADI	acceptable daily intake
ALP	alkaline phosphatase
ALT	alanine aminotransferase
AST	aspartate aminotransferase
ATP	adenosine triphosphate
ATZ	atrazine
Bax	Bcl-2-associated X
Bcl-2	B-cell lymphoma-2
BPA	bisphenol A
BPAF	bisphenol AF
BPF	bisphenol F
BPS	bisphenol S
BPs	bisphenols
BuPB	butylparaben
BW	body weight
CAT	catalase
CoA	coenzyme A
COX	cyclooxygenase
COX-2	cyclooxygenase-2
COX5B	cytochrome c oxidase subunit 5B mitochondrial
COQ9	ubiquinone biosynthesis protein COQ9_mitochondrial
DACT	diaminochlorotriazine
DDE	dichlorodiphenyldichloroethylene
DEHP	di-(2-ethylhexyl) phthalate
DES	diethylstilbestrol
DNA	deoxyribonucleic acid
EDCs	endocrine-disruptive chemicals
ERK	extracellular signal-regulated kinase
ETS	electron transport system
F1	first-generation adult mice offspring
F2	second-generation adult mice offspring
GPx	glutathione peroxidase
GPx3	glutathione peroxidase 3
GR	glutathione reductase
GSH	reduced glutathione
GSHPx	glutathione peroxidase
GSIS	glucose-stimulated insulin secretion
GSSG	glutathione disulfide
GST	glutathione-S-transferase
H_2_O_2_	hydrogen peroxide
HFCCD	high-fat/high-cholesterol/high-cholic acid diet
HFD	high-fat diet
HSC	hepatic stellate cells
IL-1β	interleukin-1β
IL-6	interleukin-6
iNOS	inducible nitric oxide synthase
JAK/STAT	Janus kinase/signal transduction and transcription activation
L-NAME	N-nitro-L-arginine methyl ester
LPO	lipid peroxidation
MAFLD	metabolic-associated fatty liver disease
MAPK	mitogen-activated protein kinase
MDA	malondialdehyde
MDC	metabolism-disrupting chemicals
MEHP	mono(2-ethylhexyl) phthalate
MePB	methylparaben
mtDNA	mitochondrial DNA
MMP	mitochondrial membrane potential
MRC	mitochondrial respiratory chain
NAC	N-acetylcysteine
NAFLD	non-alcoholic fatty liver disease
NF-κB	nuclear factor kappa-B
NO	nitric oxide
NOAEL	no observed adverse effect level
NP	nonylphenol
Nrf1	nuclear respiratory factor 1
Nrf2	nuclear factor erythroid 2-related factor factor 2
NTP	National Toxicology Program
OCR	oxygen consumption rate
Ogdh	oxoglutarate degydrogenase
OH-PCBs	hydroxylated PCBs
OP	octylphenol
OXPHOS	oxidative phosphorylation
PARP-1	poly [ADP-ribose] polymerase 1
PCBs	polychlorinated biphenyls
PCDEs	polychlorinated diphenyl ethers
PFOA	perfluorooctanoic acid
PFOS	perfluorooctane sulfonate
PGC-1α	peroxisome proliferator-activated receptor-gamma coactivator 1α
PKC	protein kinase C
PND6	postnatal day 6
POD	peroxidase
PrPB	propylparaben
RNS	reactive nitrogen species
ROS	reactive oxygen species
Sdhb	succinate dehydrogenase iron-sulfur subunit
SOD	superoxide dismutase
SIRT1	sirtuin-1
SIRT3	sirtuin-3
T-AOC	total antioxidant capacity
TBARS	thiobarbituric acid reactive substances
TBT	trybutyltin
TCDD	2,3,7,8-tetrachlorodibenzo-p-dioxin
TCS	triclosan
TFAM	mitochondrial transcription factor A
TPP	triphenylphosphate
TDI	tolerable daily intake
TNF-α	tumor necrosis factor-α
UCP2	uncoupling protein 2

## References

[1] Lopez-Jimenez F., Almahmeed W., Bays H., Cuevas A., Di Angelantonio E., le Roux C.W., Sattar N., Sun M.C., Wittert G., Pinto F.J. (2022). Obesity and cardiovascular disease: Mechanistic insights and management strategies. A joint position paper by the World Heart Federation and World Obesity Federation. Eur. J. Prev. Cardiol..

[2] Preda A., Carbone F., Tirandi A., Montecucco F., Liberale L. (2023). Obesity phenotypes and cardiovascular risk: From pathophysiology to clinical management. Rev. Endocr. Metab. Disord..

[3] Schwartz M.W., Seeley R.J., Zeltser L.M., Drewnowski A., Ravussin E., Redman L.M., Leibel R.L. (2017). Obesity Pathogenesis: An Endocrine Society Scientific Statement. Endocr. Rev..

[4] Papalou O., Kandaraki E.A., Papadakis G., Diamanti-Kandarakis E. (2019). Endocrine Disrupting Chemicals: An Occult Mediator of Metabolic Disease. Front. Endocrinol..

[5] Brown R.E., Sharma A.M., Ardern C.I., Mirdamadi P., Mirdamadi P., Kuk J.L. (2016). Secular differences in the association between caloric intake, macronutrient intake, and physical activity with obesity. Obes. Res. Clin. Pract..

[6] Neel B.A., Sargis R.M. (2011). The Paradox of Progress: Environmental Disruption of Metabolism and the Diabetes Epidemic. Diabetes.

[7] Baillie-Hamilton P.F. (2002). Chemical Toxins: A Hypothesis to Explain the Global Obesity Epidemic. J. Altern. Complement. Med..

[8] Schnegelberger R.D., Lang A.L., Arteel G.E., Beier J.I. (2021). Environmental toxicant-induced maladaptive mitochondrial changes: A potential unifying mechanism in fatty liver disease?. Acta Pharm. Sin. B.

[9] Colborn T., Saal F.S.V., Soto A.M. (1993). Developmental effects of endocrine-disrupting chemicals in wildlife and humans. Environ. Health Perspect..

[10] Grun F., Blumberg B. (2006). Environmental Obesogens: Organotins and Endocrine Disruption via Nuclear Receptor Signaling. Endocrinology.

[11] Heindel J.J., Blumberg B. (2019). Environmental Obesogens: Mechanisms and Controversies. Annu. Rev. Pharmacol. Toxicol..

[12] La Merrill M., Birnbaum L.S. (2011). Childhood Obesity and Environmental Chemicals. Mt. Sinai J. Med..

[13] Heindel J.J., Blumberg B., Cave M., Machtinger R., Mantovani A., Mendez M.A., Nadal A., Palanza P., Panzica G., Sargis R. (2017). Metabolism disrupting chemicals and metabolic disorders. Reprod. Toxicol..

[14] Mosca A., Manco M., Braghini M.R., Cianfarani S., Maggiore G., Alisi A., Vania A. (2024). Environment, Endocrine Disruptors, and Fatty Liver Disease Associated with Metabolic Dysfunction (MASLD). Metabolites.

[15] Fritsche K., Ziková-Kloas A., Marx-Stoelting P., Braeuning A. (2023). Metabolism-Disrupting Chemicals Affecting the Liver: Screening, Testing, and Molecular Pathway Identification. Int. J. Mol. Sci..

[16] Amato A.A., Wheeler H.B., Blumberg B. (2021). Obesity and endocrine-disrupting chemicals. Endocr. Connect..

[17] Hinault C., Caroli-Bosc P., Bost F., Chevalier N. (2023). Critical Overview on Endocrine Disruptors in Diabetes Mellitus. Int. J. Mol. Sci..

[18] Predieri B., Bruzzi P., Bigi E., Ciancia S., Madeo S.F., Lucaccioni L., Iughetti L. (2020). Endocrine Disrupting Chemicals and Type 1 Diabetes. Int. J. Mol. Sci..

[19] Massart J., Begriche K., Corlu A., Fromenty B. (2022). Xenobiotic-Induced Aggravation of Metabolic-Associated Fatty Liver Disease. Int. J. Mol. Sci..

[20] Reddam A., McLarnan S., Kupsco A. (2022). Environmental Chemical Exposures and Mitochondrial Dysfunction: A Review of Recent Literature. Curr. Environ. Health Rep..

[21] Meyer J.N., Chan S.S. (2017). Sources, mechanisms, and consequences of chemical-induced mitochondrial toxicity. Toxicology.

[22] Blajszczak C., Bonini M.G. (2017). Mitochondria targeting by environmental stressors: Implications for redox cellular signaling. Toxicology.

[23] Leung M.C., Meyer J.N. (2019). Mitochondria as a target of organophosphate and carbamate pesticides: Revisiting common mechanisms of action with new approach methodologies. Reprod. Toxicol..

[24] Bianchi S., Nottola S.A., Torge D., Palmerini M.G., Necozione S., Macchiarelli G. (2020). Association between Female Reproductive Health and Mancozeb: Systematic Review of Experimental Models. Int. J. Environ. Res. Public Health.

[25] Chen T., Tan J., Wan Z., Zou Y., Afewerky H.K., Zhang Z., Zhang T. (2017). Effects of Commonly Used Pesticides in China on the Mitochondria and Ubiquitin-Proteasome System in Parkinson’s Disease. Int. J. Mol. Sci..

[26] Monzel A.S., Enríquez J.A., Picard M. (2023). Multifaceted mitochondria: Moving mitochondrial science beyond function and dysfunction. Nat. Metab..

[27] Picard M., Shirihai O.S. (2022). Mitochondrial signal transduction. Cell Metab..

[28] Mourokh L., Friedman J. (2024). Mitochondria at the Nanoscale: Physics Meets Biology—What Does It Mean for Medicine?. Int. J. Mol. Sci..

[29] Bleier L., Wittig I., Heide H., Steger M., Brandt U., Dröse S. (2015). Generator-specific targets of mitochondrial reactive oxygen species. Free Radic. Biol. Med..

[30] Muntean D.M., Sturza A., Dănilă M.D., Borza C., Duicu O.M., Mornoș C. (2016). The Role of Mitochondrial Reactive Oxygen Species in Cardiovascular Injury and Protective Strategies. Oxidative Med. Cell. Longev..

[31] Hartsoe P., Holguin F., Chu H.W. (2024). Mitochondrial Dysfunction and Metabolic Reprogramming in Obesity and Asthma. Int. J. Mol. Sci..

[32] Li C.-L., Liu J.-F., Liu S.-F. (2024). Mitochondrial Dysfunction in Chronic Obstructive Pulmonary Disease: Unraveling the Molecular Nexus. Biomedicines.

[33] Marroqui L., Tudurí E., Alonso-Magdalena P., Quesada I., Nadal Á., dos Santos R.S. (2018). Mitochondria as target of endocrine-disrupting chemicals: Implications for type 2 diabetes. J. Endocrinol..

[34] Chen J., Stimpson S.E., Fernandez-Bueno G.A., Mathews C.E. (2018). Mitochondrial Reactive Oxygen Species and Type 1 Diabetes. Antioxid. Redox Signal..

[35] Avram V.F., Merce A.P., Hâncu I.M., Bătrân A.D., Kennedy G., Rosca M.G., Muntean D.M. (2022). Impairment of Mitochondrial Respiration in Metabolic Diseases: An Overview. Int. J. Mol. Sci..

[36] Antonucci S., Di Lisa F., Kaludercic N. (2021). Mitochondrial reactive oxygen species in physiology and disease. Cell Calcium.

[37] Nickel A., Kohlhaas M., Maack C. (2014). Mitochondrial reactive oxygen species production and elimination. J. Mol. Cell. Cardiol..

[38] Huang Z., Chen Y., Zhang Y. (2020). Mitochondrial reactive oxygen species cause major oxidative mitochondrial DNA damages and repair pathways. J. Biosci..

[39] Corkey B.E. (2023). Reactive oxygen species: Role in obesity and mitochondrial energy efficiency. Philos. Trans. R. Soc. B Biol. Sci..

[40] Heindel J.J., Lustig R.H., Howard S., Corkey B.E. (2024). Obesogens: A unifying theory for the global rise in obesity. Int. J. Obes..

[41] Fu H., Ge Y., Liu X., Deng S., Li J., Tan P., Yang Y., Wu Z. (2024). Exposure to the environmental pollutant chlorpyrifos induces hepatic toxicity through activation of the JAK/STAT and MAPK pathways. Sci. Total. Environ..

[42] Han C., Sheng J., Pei H., Sheng Y., Wang J., Zhou X., Li W., Cao C., Yang Y. (2023). Environmental toxin chlorpyrifos induces liver injury by activating P53-mediated ferroptosis via GSDMD-mtROS. Ecotoxicol. Environ. Saf..

[43] Ahn C., Jeung E.-B. (2023). Endocrine-Disrupting Chemicals and Disease Endpoints. Int. J. Mol. Sci..

[44] Bergman Å., Heindel J.J., Kasten T., Kidd K.A., Jobling S., Neira M., Zoeller R.T., Becher G., Bjerregaard P., Bornman R. (2013). The Impact of Endocrine Disruption: A Consensus Statement on the State of the Science. Environ. Health Perspect..

[45] Vandenberg L.N., Maffini M.V., Sonnenschein C., Rubin B.S., Soto A.M. (2009). Bisphenol-A and the Great Divide: A Review of Controversies in the Field of Endocrine Disruption. Endocr. Rev..

[46] Khan N.G., Tungekar B., Adiga D., Chakrabarty S., Rai P.S., Kabekkodu S.P. (2023). Alterations induced by Bisphenol A on cellular organelles and potential relevance on human health. Biochim. Biophys. Acta Mol. Cell Res..

[47] Bindhumol V., Chitra K., Mathur P. (2003). Bisphenol A induces reactive oxygen species generation in the liver of male rats. Toxicology.

[48] Lang I.A., Galloway T.S., Scarlett A., Henley W.E., Depledge M., Wallace R.B., Melzer D. (2008). Association of Urinary Bisphenol A Concentration with Medical Disorders and Laboratory Abnormalities in Adults. JAMA.

[49] Rancière F., Lyons J.G., Loh V.H., Botton J., Galloway T., Wang T., Shaw J.E., Magliano D.J. (2015). Bisphenol A and the risk of cardiometabolic disorders: A systematic review with meta-analysis of the epidemiological evidence. Environ. Health.

[50] Huc L., Lemarié A., Guéraud F., Héliès-Toussaint C. (2012). Low concentrations of bisphenol A induce lipid accumulation mediated by the production of reactive oxygen species in the mitochondria of HepG2 cells. Toxicol. In Vitro.

[51] Moon M.K., Kim M.J., Jung I.K., Koo Y.D., Ann H.Y., Lee K.J., Kim S.H., Yoon Y.C., Cho B.-J., Park K.S. (2012). Bisphenol A Impairs Mitochondrial Function in the Liver at Doses below the No Observed Adverse Effect Level. J. Korean Med. Sci..

[52] Hassan Z.K., Elobeid M.A., Virk P., Omer S.A., ElAmin M., Daghestani M.H., AlOlayan E.M. (2012). Bisphenol A Induces Hepatotoxicity through Oxidative Stress in Rat Model. Oxidative Med. Cell. Longev..

[53] Khan S., Beigh S., Chaudhari B.P., Sharma S., Abdi S.A.H., Ahmad S., Ahmad F., Parvez S., Raisuddin S. (2015). Mitochondrial dysfunction induced by Bisphenol A is a factor of its hepatotoxicity in rats. Environ. Toxicol..

[54] Kourouma A., Quan C., Duan P., Qi S., Yu T., Wang Y., Yang K. (2015). Bisphenol A Induces Apoptosis in Liver Cells through Induction of ROS. Adv. Toxicol..

[55] Hassani F.V., Abnous K., Mehri S., Jafarian A., Birner-Gruenberger R., Robati R.Y., Hosseinzadeh H. (2018). Proteomics and phosphoproteomics analysis of liver in male rats exposed to bisphenol A: Mechanism of hepatotoxicity and biomarker discovery. Food Chem. Toxicol..

[56] Wang K., Zhao Z., Ji W. (2019). Bisphenol A induces apoptosis, oxidative stress and inflammatory response in colon and liver of mice in a mitochondria-dependent manner. Biomed. Pharmacother..

[57] Eweda S.M., Newairy A.S.A., Abdou H.M., Gaber A.S. (2020). Bisphenol A-induced oxidative damage in the hepatic and cardiac tissues of rats: The modulatory role of sesame lignans. Exp. Ther. Med..

[58] Pirozzi C., Lama A., Annunziata C., Cavaliere G., Ruiz-Fernandez C., Monnolo A., Comella F., Gualillo O., Stornaiuolo M., Mollica M.P. (2020). Oral Bisphenol A Worsens Liver Immune-Metabolic and Mitochondrial Dysfunction Induced by High-Fat Diet in Adult Mice: Cross-Talk between Oxidative Stress and Inflammasome Pathway. Antioxidants.

[59] Lee J.-L., Wang Y.-C., Hsu Y.-A., Chen C.-S., Weng R.-C., Lu Y.-P., Chuang C.-Y., Wan L. (2022). Bisphenol A Coupled with a High-Fat Diet Promotes Hepatosteatosis through Reactive-Oxygen-Species-Induced CD36 Overexpression. Toxics.

[60] Liu R., Liu B., Tian L., Jiang X., Li X., Cai D., Sun J., Bai W., Jin Y. (2022). Exposure to Bisphenol A Caused Hepatoxicity and Intestinal Flora Disorder in Rats. Int. J. Mol. Sci..

[61] Jiang Y., Xia W., Zhu Y., Li X., Wang D., Liu J., Chang H., Li G., Xu B., Chen X. (2014). Mitochondrial dysfunction in early life resulted from perinatal bisphenol A exposure contributes to hepatic steatosis in rat offspring. Toxicol. Lett..

[62] Linillos-Pradillo B., Rancan L., Paredes S.D., Schlumpf M., Lichtensteiger W., Vara E., Tresguerres J.F. (2023). Low Dose of BPA Induces Liver Injury through Oxidative Stress, Inflammation and Apoptosis in Long–Evans Lactating Rats and Its Perinatal Effect on Female PND6 Offspring. Int. J. Mol. Sci..

[63] Qiu W., Chen J., Li Y., Chen Z., Jiang L., Yang M., Wu M. (2016). Oxidative stress and immune disturbance after long-term exposure to bisphenol A in juvenile common carp (*Cyprinus carpio*). Ecotoxicol. Environ. Saf..

[64] Afzal G., Ahmad H.I., Hussain R., Jamal A., Kiran S., Hussain T., Saeed S., Nisa M.U. (2022). Bisphenol A Induces Histopathological, Hematobiochemical Alterations, Oxidative Stress, and Genotoxicity in Common Carp (*Cyprinus carpio* L.). Oxidative Med. Cell. Longev..

[65] Zhang H., Yang R., Shi W., Zhou X., Sun S. (2021). The association between bisphenol A exposure and oxidative damage in rats/mice: A systematic review and meta-analysis. Environ. Pollut..

[66] Ijaz M.U., Shahab M.S., Samad A., Ashraf A., Al-Ghanim K., Mruthinti S.S., Mahboob S. (2022). Tangeretin ameliorates bisphenol induced hepatocyte injury by inhibiting inflammation and oxidative stress. Saudi J. Biol. Sci..

[67] Nagarajan M., Maadurshni G.B., Manivannan J. (2024). Bisphenol A (BPA) exposure aggravates hepatic oxidative stress and inflammatory response under hypertensive milieu—Impact of low dose on hepatocytes and influence of MAPK and ER stress pathways. Food Chem. Toxicol..

[68] Meng Z., Tian S., Yan J., Jia M., Yan S., Li R., Zhang R., Zhu W., Zhou Z. (2019). Effects of perinatal exposure to BPA, BPF and BPAF on liver function in male mouse offspring involving in oxidative damage and metabolic disorder. Environ. Pollut..

[69] Azevedo L.F., Carneiro M.F.H., Dechandt C.R.P., Cassoli J.S., Alberici L.C., Barbosa F. (2020). Global liver proteomic analysis of Wistar rats chronically exposed to low-levels of bisphenol A and S. Environ. Res..

[70] Zhang R., Liu R., Zong W. (2016). Bisphenol S Interacts with Catalase and Induces Oxidative Stress in Mouse Liver and Renal Cells. J. Agric. Food Chem..

[71] Liu K., Kadannagari S., Deruiter J., Pathak S., Abbott K.L., Salamat J.M., Pondugula S.R., Akingbemi B.T., Dhanasekaran M. (2023). Effects of developmental exposures to Bisphenol-A and Bisphenol-S on hepatocellular function in male Long-Evans rats. Life Sci..

[72] Mahim S.S., Anjali V.R., Remya V.S., Reshmi S., Devi C.A. (2021). Oxidative stress responses of a freshwater fish, *Labeo rohita*, to a xenobiotic, bisphenol S. J. Biochem. Mol. Toxicol..

[73] Alharbi H.F., Algonaiman R., Alduwayghiri R., Aljutaily T., Algheshairy R.M., Almutairi A.S., Alharbi R.M., Alfurayh L.A., Alshahwan A.A., Alsadun A.F. (2022). Exposure to Bisphenol A Substitutes, Bisphenol S and Bisphenol F, and Its Association with Developing Obesity and Diabetes Mellitus: A Narrative Review. Int. J. Environ. Res. Public Health.

[74] Štefunková N., Greifová H., Jambor T., Tokárová K., Zuščíková L., Bažány D., Massányi P., Capcarová M., Lukáč N. (2023). Comparison of the Effect of BPA and Related Bisphenols on Membrane Integrity, Mitochondrial Activity, and Steroidogenesis of H295R Cells In Vitro. Life.

[75] Shi R., Liu Z., Liu T. (2021). The antagonistic effect of bisphenol A and nonylphenol on liver and kidney injury in rats. Immunopharmacol. Immunotoxicol..

[76] Mukherjee U., Samanta A., Biswas S., Ghosh S., Das S., Banerjee S., Maitra S. (2022). Chronic exposure to nonylphenol induces oxidative stress and liver damage in male zebrafish (*Danio rerio*): Mechanistic insight into cellular energy sensors, lipid accumulation and immune modulation. Chem. Interact..

[77] Espina C., Straif K., Friis S., Kogevinas M., Saracci R., Vainio H., Schüz J. (2015). European Code against Cancer 4th Edition: Environment, occupation and cancer. Cancer Epidemiol..

[78] McCann M.S., Fernandez H.R., Flowers S.A., Maguire-Zeiss K.A. (2021). Polychlorinated biphenyls induce oxidative stress and metabolic responses in astrocytes. Neurotoxicology.

[79] Gupta P., Thompson B.L., Wahlang B., Jordan C.T., Hilt J.Z., Hennig B., Dziubla T. (2017). The environmental pollutant, polychlorinated biphenyls, and cardiovascular disease: A potential target for antioxidant nanotherapeutics. Drug Deliv. Transl. Res..

[80] Dhakal K., Gadupudi G.S., Lehmler H.-J., Ludewig G., Duffel M.W., Robertson L.W. (2018). Sources and toxicities of phenolic polychlorinated biphenyls (OH-PCBs). Environ. Sci. Pollut. Res..

[81] VanEtten S.L., Bonner M.R., Ren X., Birnbaum L.S., Kostyniak P.J., Wang J., Olson J.R. (2021). Effect of exposure to 2,3,7,8-Tetrachlorodibenzo-p-dioxin (TCDD) and polychlorinated biphenyls (PCBs) on mitochondrial DNA (mtDNA) copy number in rats. Toxicology.

[82] Van Den Berg M., Birnbaum L.S., Denison M., De Vito M., Farland W., Feeley M., Fiedler H., Håkansson H., Hanberg A., Haws L. (2006). The 2005 World Health Organization Reevaluation of Human and Mammalian Toxic Equivalency Factors for Dioxins and Dioxin-Like Compounds. Toxicol. Sci..

[83] (2006). National Toxicology Program. Toxicology and carcinogenesis studies of a binary mixture of 3,3′,4,4′,5-pentachlorobiphenyl (PCB 126) (Cas No. 57465-28-8) and 2,3′,4,4′,5-pentachlorobiphenyl (PCB 118) (Cas No. 31508-00-6) in female Harlan Sprague-Dawley rats (gavage studies). Natl. Toxicol. Program Tech. Rep. Ser..

[84] Ounnas F., Privé F., Lamarche F., Salen P., Favier-Hininger I., Marchand P., Le Bizec B., Venisseau A., Batandier C., Fontaine E. (2016). A relevant exposure to a food matrix contaminated environmentally by polychlorinated biphenyls induces liver and brain disruption in rats. Chemosphere.

[85] Deng P., Barney J., Petriello M.C., Morris A.J., Wahlang B., Hennig B. (2019). Hepatic metabolomics reveals that liver injury increases PCB 126-induced oxidative stress and metabolic dysfunction. Chemosphere.

[86] Jin Y., Wang L., Chen G., Lin X., Miao W., Fu Z. (2014). Exposure of mice to atrazine and its metabolite diaminochlorotriazine elicits oxidative stress and endocrine disruption. Environ. Toxicol. Pharmacol..

[87] Sagarkar S., Gandhi D., Devi S.S., Sakharkar A., Kapley A. (2016). Atrazine exposure causes mitochondrial toxicity in liver and muscle cell lines. Indian J. Pharmacol..

[88] Halden R.U. (2010). Plastics and Health Risks. Annu. Rev. Public Health.

[89] Lee K.-I., Chiang C.-W., Lin H.-C., Zhao J.-F., Li C.-T., Shyue S.-K., Lee T.-S. (2016). Maternal exposure to di-(2-ethylhexyl) phthalate exposure deregulates blood pressure, adiposity, cholesterol metabolism and social interaction in mouse offspring. Arch. Toxicol..

[90] Zhao J.-F., Hsiao S.-H., Hsu M.-H., Pao K.-C., Kou Y.R., Shyue S.-K., Lee T.-S. (2016). Di-(2-ethylhexyl) phthalate accelerates atherosclerosis in apolipoprotein E-deficient mice. Arch. Toxicol..

[91] Chen H., Zhang W., Rui B.-B., Yang S.-M., Xu W.-P., Wei W. (2016). Di(2-ethylhexyl) phthalate exacerbates non-alcoholic fatty liver in rats and its potential mechanisms. Environ. Toxicol. Pharmacol..

[92] Tickner J.A., Schettler T., Guidotti T., McCally M., Rossi M. (2001). Health risks posed by use of Di-2-ethylhexyl phthalate (DEHP) in PVC medical devices: A critical review. Am. J. Ind. Med..

[93] Lee C.-Y., Suk F.-M., Twu Y.-C., Liao Y.-J. (2020). Long-Term Exposure to Low-Dose Di-(2-ethylhexyl) Phthalate Impairs Cholesterol Metabolism in Hepatic Stellate Cells and Exacerbates Liver Fibrosis. Int. J. Environ. Res. Public Health.

[94] Li G., Zhao C.-Y., Wu Q., Guan S.-Y., Jin H.-W., Na X.-L., Zhang Y.-B. (2021). Integrated metabolomics and transcriptomics reveal di(2-ethylhexyl) phthalate-induced mitochondrial dysfunction and glucose metabolism disorder through oxidative stress in rat liver. Ecotoxicol. Environ. Saf..

[95] Zhang Q., Zhao Y., Talukder M., Han Y., Zhang C., Li X.-N., Li J.-L. (2019). Di(2-ethylhexyl) phthalate induced hepatotoxicity in quail (*Coturnix japonica*) via modulating the mitochondrial unfolded protein response and NRF2 mediated antioxidant defense. Sci. Total. Environ..

[96] Park C.G., Sung B., Ryu C.S., Kim Y.J. (2020). Mono-(2-ethylhexyl) phthalate induces oxidative stress and lipid accumulation in zebrafish liver cells. Comp. Biochem. Physiol. C Toxicol. Pharmacol..

[97] Han R., Hu M., Zhong Q., Wan C., Liu L., Li F., Zhang F., Ding W. (2018). Perfluorooctane sulphonate induces oxidative hepatic damage via mitochondria-dependent and NF-kappa B/TNF-alpha-mediated pathway. Chemosphere.

[98] Domingo J.L. (2006). Polychlorinated diphenyl ethers (PCDEs): Environmental levels, toxicity and human exposure: A review of the published literature. Environ. Int..

[99] Ye C., Xiong W., Shi S., Shi J., Yang W., Zhang X. (2022). Biomarker Responses, Gene Expression Alterations, and Histological Changes in Zebrafish (*Danio rerio*) after in vivo Exposure to Polychlorinated Diphenyl Ethers. Front. Physiol..

[100] Margina D., Nițulescu G.M., Ungurianu A., Mesnage R., Goumenou M., Sarigiannis D.A., Aschner M., Spandidos D.A., Renieri E.A., Tsatsakis A. (2019). Overview of the effects of chemical mixtures with endocrine disrupting activity in the context of real-life risk simulation: An integrative approach (Review). World Acad. Sci. J..

[101] Vardakas P., Veskoukis A.S., Rossiou D., Gournikis C., Kapetanopoulou T., Karzi V., Docea A.O., Tsatsakis A., Kouretas D. (2022). A Mixture of Endocrine Disruptors and the Pesticide Roundup^®^ Induce Oxidative Stress in Rabbit Liver When Administered under the Long-Term Low-Dose Regimen: Reinforcing the Notion of Real-Life Risk Simulation. Toxics.

[102] Tsatsakis A., Goumenou M., Liesivuori J., Dekant W., Hernández A.F. (2019). Toxicology for real-life risk simulation—Editorial preface to this special issue. Toxicol. Lett..

[103] Xia W., Jiang Y., Li Y., Wan Y., Liu J., Ma Y., Mao Z., Chang H., Li G., Xu B. (2014). Early-Life Exposure to Bisphenol A Induces Liver Injury in Rats Involvement of Mitochondria-Mediated Apoptosis. PLoS ONE.

[104] Mornagui B., Rezg R., Repond C., Pellerin L. (2022). Bisphenol S favors hepatic steatosis development via an upregulation of liver MCT1 expression and an impairment of the mitochondrial respiratory system. J. Cell. Physiol..

[105] Kim K. (2024). The Role of Endocrine Disruption Chemical-Regulated Aryl Hydrocarbon Receptor Activity in the Pathogenesis of Pancreatic Diseases and Cancer. Int. J. Mol. Sci..

[106] Wei J., Lin Y., Li Y., Ying C., Chen J., Song L., Zhou Z., Lv Z., Xia W., Chen X. (2011). Perinatal Exposure to Bisphenol A at Reference Dose Predisposes Offspring to Metabolic Syndrome in Adult Rats on a High-Fat Diet. Endocrinology.

[107] Carchia E., Porreca I., Almeida P.J., D’Angelo F., Cuomo D., Ceccarelli M., De Felice M., Mallardo M., Ambrosino C. (2015). Evaluation of low doses BPA-induced perturbation of glycemia by toxicogenomics points to a primary role of pancreatic islets and to the mechanism of toxicity. Cell Death Dis..

[108] Susiarjo M., Xin F., Bansal A., Stefaniak M., Li C., Simmons R.A., Bartolomei M.S. (2015). Bisphenol A Exposure Disrupts Metabolic Health across Multiple Generations in the Mouse. Endocrinology.

[109] Bansal A., Rashid C., Xin F., Li C., Polyak E., Duemler A., van der Meer T., Stefaniak M., Wajid S., Doliba N. (2017). Sex- and Dose-Specific Effects of Maternal Bisphenol A Exposure on Pancreatic Islets of First- and Second-Generation Adult Mice Offspring. Environ. Health Perspect..

[110] Moon M.K., Jeong I.-K., Oh T.J., Ahn H.Y., Kim H.H., Park Y.J., Jang H.C., Park K.S. (2015). Long-term oral exposure to bisphenol A induces glucose intolerance and insulin resistance. J. Endocrinol..

[111] Song L., Xia W., Zhou Z., Li Y., Lin Y., Wei J., Wei Z., Xu B., Shen J., Li W. (2012). Low-level phenolic estrogen pollutants impair islet morphology and β-cell function in isolated rat islets. J. Endocrinol..

[112] Li X., Zhou L., Ni Y., Wang A., Hu M., Lin Y., Hong C., Wan J., Chen B., Fang L. (2017). Nonylphenol induces pancreatic damage in rats through mitochondrial dysfunction and oxidative stress. Toxicol. Res..

[113] National Center for Biotechnology Information (2024). PubChem Compound Summary for CID 3032732, Tributyl tin. https://pubchem.ncbi.nlm.nih.gov/compound/Tributyl-tin.

[114] Chen Y.-W., Lan K.-C., Tsai J.-R., Weng T.-I., Yang C.-Y., Liu S.-H. (2017). Tributyltin exposure at noncytotoxic doses dysregulates pancreatic β-cell function in vitro and in vivo. Arch. Toxicol..

[115] Dos Santos R.S., Babiloni-Chust I., Marroqui L., Nadal A. (2022). Screening of Metabolism-Disrupting Chemicals on Pancreatic α-Cells Using In Vitro Methods. Int. J. Mol. Sci..

[116] Makaji E., Raha S., Wade M.G., Holloway A.C. (2011). Effect of Environmental Contaminants on Beta Cell Function. Int. J. Toxicol..

[117] Jaacks L.M., Vandevijvere S., Pan A., McGowan C.J., Wallace C., Imamura F., Mozaffarian D., Swinburn B., Ezzati M. (2019). The obesity transition: Stages of the global epidemic. Lancet Diabetes Endocrinol..

[118] Küblbeck J., Vuorio T., Niskanen J., Fortino V., Braeuning A., Abass K., Rautio A., Hakkola J., Honkakoski P., Levonen A.-L. (2020). The EDCMET Project: Metabolic Effects of Endocrine Disruptors. Int. J. Mol. Sci..

[119] Ribeiro E., Ladeira C., Viegas S. (2017). EDCs Mixtures: A Stealthy Hazard for Human Health?. Toxics.

[120] Duh-Leong C., Maffini M.V., Kassotis C.D., Vandenberg L.N., Trasande L. (2023). The regulation of endocrine-disrupting chemicals to minimize their impact on health. Nat. Rev. Endocrinol..

